# A Case Report of Acute Pericarditis Presenting With Acute Aortic Dissection

**DOI:** 10.7759/cureus.73818

**Published:** 2024-11-16

**Authors:** Pamella Morello, Sahar S Abdelmoneim, Patricia D Armas Gonzalez, Santiago Pastori, Sabas Gomez

**Affiliations:** 1 Internal Medicine, Dr. Kiran C. Patel College of Osteopathic Medicine, Nova Southeastern University, Fort Lauderdale, USA; 2 Internal Medicine, Larkin Community Hospital, Hialeah, USA; 3 General Internal Medicine/Cardiovascular Medicine, Assiut University Hospital, Assiut, EGY; 4 Cardiology, Larkin Community Hospital, Hialeah, USA

**Keywords:** acute aortic syndromes, acute pericarditis, aortic aneurysm, aortic dissection syndromes, ecg changes

## Abstract

This case report presents a rare occurrence of acute pericarditis coinciding with acute aortic dissection (AAD), emphasizing the significance of recognizing and managing these dual conditions effectively. It highlights the necessity of a multidisciplinary approach and underscores the importance of maintaining a high index of suspicion regardless of symptomatology to ensure prompt patient management and complication prevention. This case describes a 91-year-old male patient, who initially presented with mild chest pain, and was later diagnosed with AAD alongside acute pericarditis. The report emphasizes the critical role of early recognition in such presentations and aims to enhance awareness among healthcare professionals and readers regarding this complex disease and its potential complications.

## Introduction

Acute aortic syndrome (AAS) is a comprehensive term employed to delineate various forms of life-threatening aortic pathology characterized by analogous clinical presentations yet divergent etiologies, pathophysiological mechanisms, and prognostic implications. The classification encapsulates aortic dissection (AD), intramural hematoma (IMH), and penetrating aortic ulcer (PAU) [1]. Acute pericarditis, on the other hand, confers lower rates of mortality and is defined as an inflammation of the outer layer of the heart known as the pericardium. Published literature has reported acute pericarditis as a masquerade for acute aortic dissection (AAD) [2].

To highlight this clinical presentation, we report a unique case of a 91-year-old male patient with mild chest pain diagnosed with AAD with coexisting acute pericarditis. The patient has provided informed consent for the same. This case report emphasizes the importance of having a high index of suspicion, regardless of symptomatology, for prompt early recognition of such presentation and help readers gain awareness of this disease with its potential complications.

## Case presentation

We present a case of a 91-year-old male patient with a pre-existing diagnosis of hyperlipidemia, hypertension, chronic obstructive pulmonary disease (COPD), and benign prostate hyperplasia noncompliant with medications. The patient presented with a persistent onset of non-pleuritic substernal chest pain (intensity rated 4/10) radiating to his left upper extremity over the preceding three days. He denied any additional symptoms apart from a history of dyspnea on exertion. The patient denied travel history, sick contact, fever, chills, shortness of breath, chest pain, palpitations, or urinary symptoms. His social history included occasional smoking and drinking alcohol on weekends and his family history was unremarkable. Additional social determinants of health encompassed unstable living conditions and a documented history of medication nonadherence. His vital signs on admission were blood pressure of 152/92 mmHg, pulse of 95 beats per minute (bpm), respiratory rate of 18 breaths/min, and oxygen saturation of 97% on room air. He was afebrile and had a body mass index (BMI) of 24.1 kg/m^2^. On evaluation, the patient was alert, and oriented to person, time, and place. Physical examination was remarkable for normal S1 and accentuated S2, with a short systolic murmur audible in the pulmonary area, with no radiation. A pulmonary exam revealed mild crackling bilaterally, with no signs of decreased breath sounds. Abdominal examination was negative for pulsating abdominal masses or bruits on auscultation. Initial electrocardiogram (ECG) revealed sinus tachycardia at 105 bpm, with nonspecific ST segment T wave changes.

The patient’s laboratory findings were significant for the parameters denoted in Table 1. Additionally, the patient was diagnosed with acute kidney injury, potentially on the background of chronic kidney disease, given the unknown renal baseline. Although troponin levels were initially negative at the emergency department, subsequent tests post-admission revealed elevated levels. He had a white blood cell (WBC) count of 5,100 cells/µL (reference range 4,500-11,000 cells/µL) and displayed a mild decrease in platelets. The serum microbiology was negative and urinalysis was unremarkable.

**Table 1:** The patient's laboratory parameters

Test	Result	Reference value/range
COVID-19	Negative	Negative
Total cholesterol (mg/dL)	209	<200
Low-density lipoprotein (LDL; mg/dL)	149	<100
Partial thromboplastin time (PTT; seconds)	41.6	25-35
D-dimer (mg/L)	2500	<0.50
Brain natriuretic peptide (BNP; pg/mL)	2110	<100
Creatinine (mg/dL)	1.54	0.7-1.3
Blood urea nitrogen (BUN; mg/dL)	27	6-24
Estimated glomerular filtration rate (eGFR; ml/min/1.73 m^2^)	43	>60
Troponin (ng/mL)	0.05	< 0.04
White blood cell (WBC) count (cells/µL)	5,100	4,500-11,000
Platelets (per µL)	124,000	150,000-450,000

**Figure 1:**
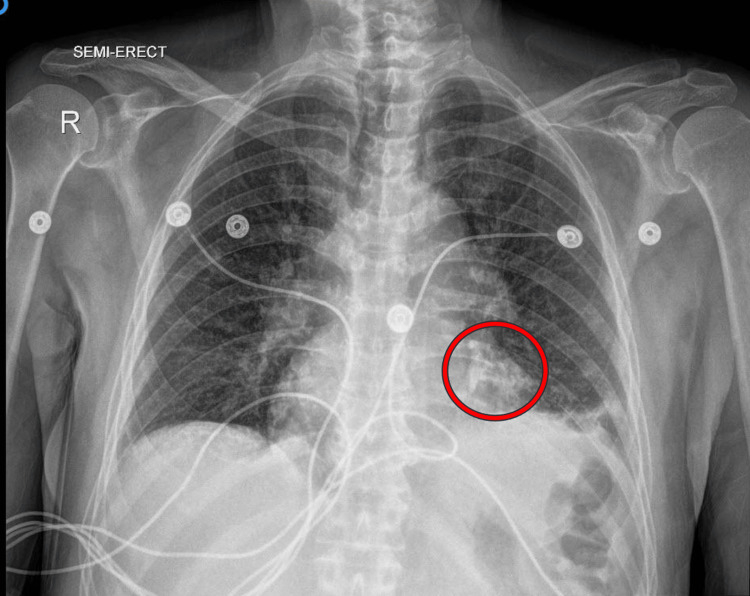
Postero-anterior (PA) chest radiography view Left basilar subsegmental atelectasis with no focal pulmonary consolidations or pleural effusions. Cardiomediastinal silhouette is within normal limits in size. Linear hyperdensity overlying the heart likely represents coronary artery calcifications (indicated by the red circle).

The chest X-ray showed a normal cardiac silhouette with no focal pulmonary consolidation or pleural effusion. However, a linear hyperdensity overlying the heart, suggestive of coronary artery calcification, was noted (Figure 1).

**Figure 2:**
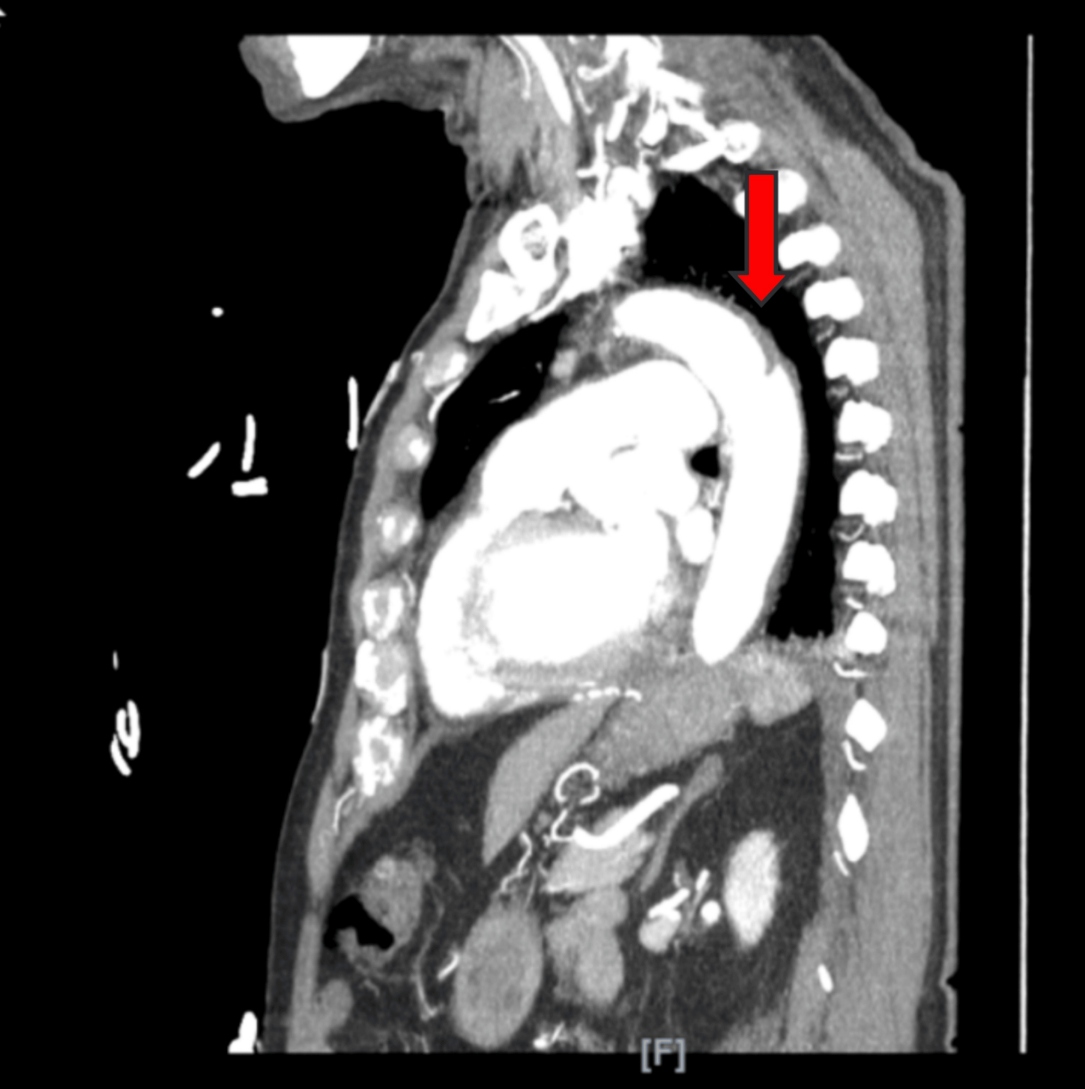
Sagittal CT cut revealing the descending aortic defect CT scan revealing AAD of the descending aorta AAD: Acute aortic dissection

**Figure 3:**
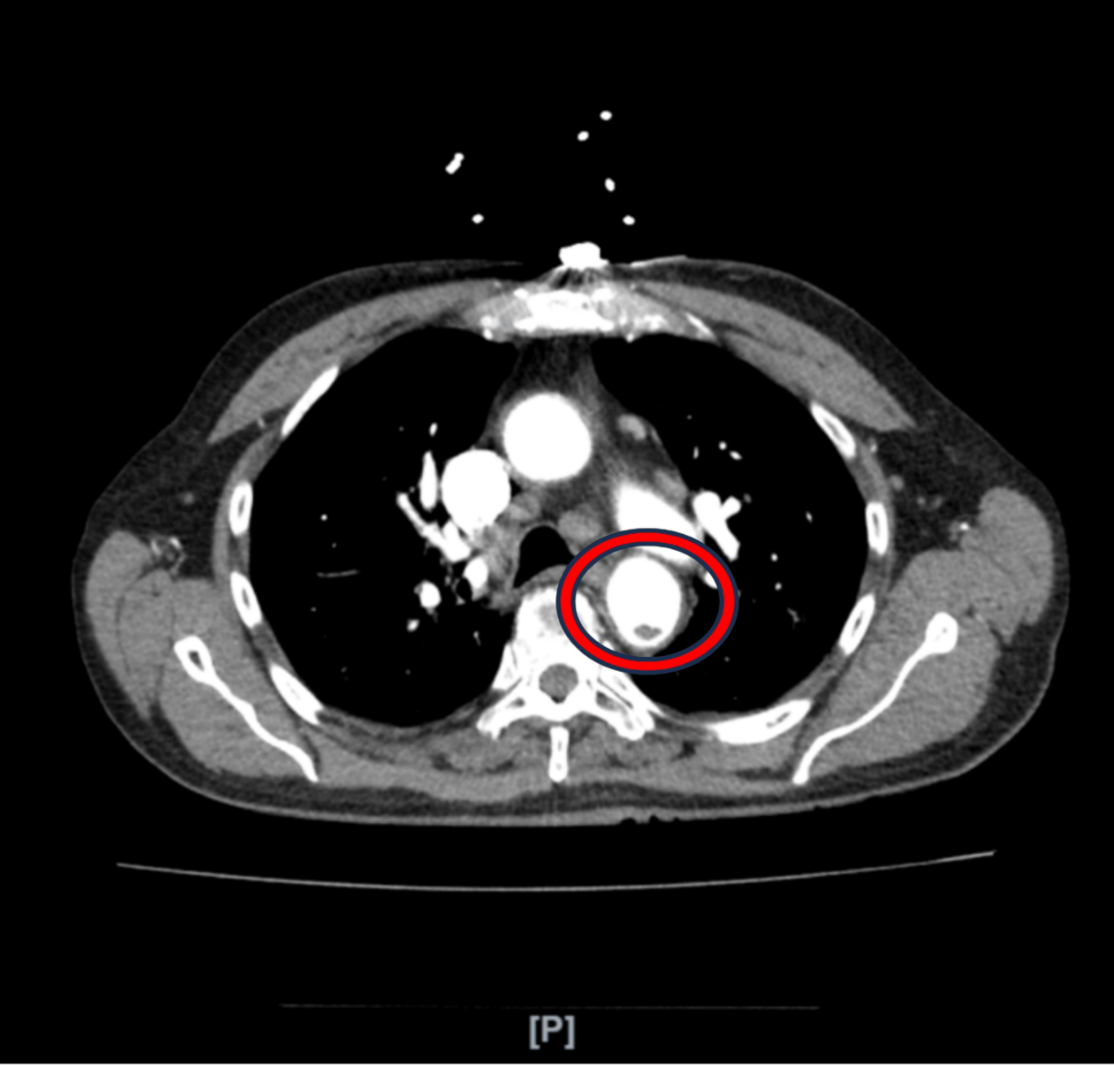
Transverse CT cut revealing a filling defect on the descending aorta The filling defect likely represents AAD. Mild pericardium effusion was also noted.

Additionally, due to elevated D-dimer and BNP levels, a computed tomography angiography (CTA) scan of the chest and aorta, following kidney protective protocol (IV fluid hydration), was performed to rule out suspicion of pulmonary embolism and aortic dissection. This decision was influenced by the patient's elevated risk factors including age, smoking, hypertension, chest pain, and elevated D-dimer levels. The test showed focal intimal irregularity of the thoracic aorta, potentially indicative of a small aortic dissection with thrombosis of the false lumen, with the internal focal intimal tear located in the descending aorta with no intramural hematoma (Figures 2, 3).

**Figure 4:**
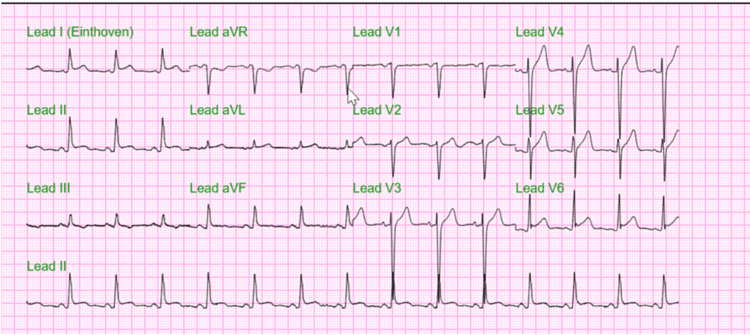
ECG revealing characteristic findings of acute pericarditis Widespread ST-segment elevation in leads V3-V6, coupled with PR-segment depression in leads I, II, V4-V6. Notably, lead V6 exhibited an ST-segment elevation ratio to T-wave amplitude exceeding 0.25 mm.

The patient was admitted to the intensive care unit for descending AAD, and rapid control of blood pressure and heart rate was initiated with esmolol 2500 mg IV drip for the first 24 hours and then substituted for oral labetalol 200 mg every eight hours as per the cardiologist's recommendation. A day after the admission, the patient continued to have chest pain. The increase in troponin levels ranged from 0.05 to 0.14 throughout the day; C-reactive protein (CRP) was 12.2 mg/dL (reference <1.0 mg/dL) and erythrocyte sedimentation rate (ESR) was 41.3 mm/hr (reference value in males<20 mm/hr). A repeat ECG revealed ST-segment elevation in the non-contiguous leads, and inferior and lateral leads, consistent with acute pericarditis (Figure 4). 

**Figure 5:**
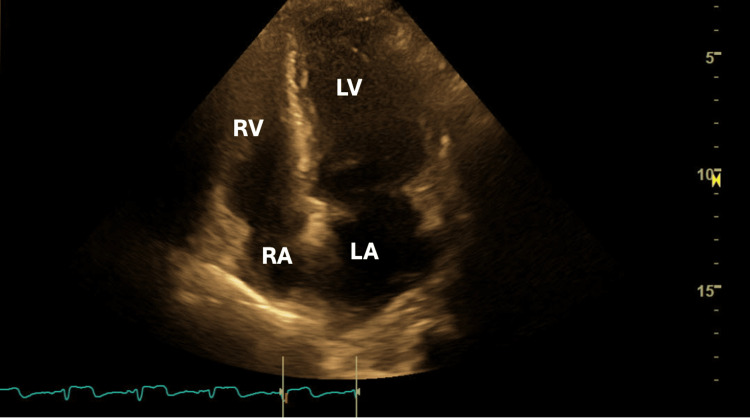
An apical four-chamber view showing the left ventricle (LV), left atrium (LA), right ventricle (RV), and right atrium (RA)

**Figure 6:**
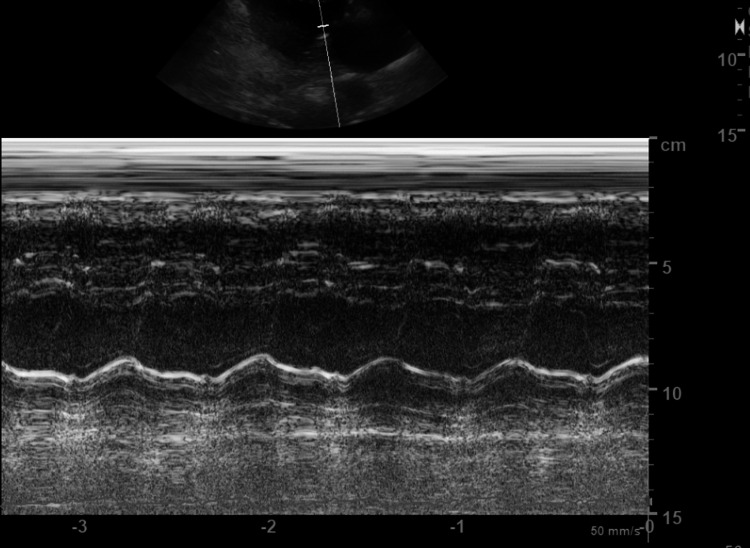
The M-mode of the left ventricle showing its preserved contractility Preserved systolic function, indicated by a left ventricular ejection fraction (LVEF) within the 55-60% range.

A transthoracic echocardiogram (TTE) was performed (Figures 5, 6). Mild to moderate concentric left ventricular hypertrophy was observed along with severe diastolic dysfunction characterized by a restrictive filling pattern. No ventricular septal defect or left ventricle thrombus was visualized. The pericardium appeared normal, not thickened and there was no pericardial effusion.

Despite a seemingly normal appearance of the pericardium on TTE, the diagnosis of acute pericarditis is established by meeting at least two of the four widely recognized criteria: pericardial chest pain, pericardial rubs detected on auscultation, new widespread ST elevation or PR depression on ECG, or the presence of a new or worsening pericardial effusion. The patient was treated empirically for acute pericarditis with oral colchicine 0.6 mg twice daily for two months, and oral ibuprofen 600 mg once daily for two weeks along with a regimen for maintaining blood pressure control. The patient symptoms improved after three days of therapy initiation and the blood pressure remained within the target goal of <120/80 mmHg. His heart rate was also under control (ranges of 60-70 bpm). The remainder of his hospital course was uneventful. He was counseled on the importance of outpatient cardiology follow-up and was discharged.

## Discussion

Our case report contributes to the limited literature on the co-occurrence of acute pericarditis and AAD. Similar to our case, Kasuhito [3] reported three instances of acute ascending aorta dissection where pericarditis was only diagnosed after repeated ECGs revealing diffuse ST elevations. The patients exhibited nonspecific symptoms of pericarditis, with each case presenting differently. Hirata et al. [4] reported 159 cases of type A AAD, where only 1.3% presented with diffuse ST-elevations, indicative of acute pericarditis. They suggested that this may result from the accumulation of blood in the pericardium due to a leak from the dissected aorta. This leads to pericardial inflammation and the observed ECG changes. In a case similar to ours, they reported a case of ascending AAD discovered on imaging despite initial normal ECG and cardiac enzyme results. Due to the persistence of the chest pain, the ECG was repeated revealing a pericarditis-like ECG pattern. The diagnosis was confirmed on CT contrast of the chest which identified mild pericardial effusion. 

The coexistence of these two conditions complicates diagnosis, increasing the risk to patients. In our case, AAD was identified first, though pericarditis is more often diagnosed before AAD due to characteristic clinical signs and testing clues such as pericarditis-like ECG changes. However, the literature highlights a range of possible presentations, from typical ECG findings of acute pericarditis (e.g., diffuse ST-elevation) without classic symptoms, to cases with friction rubs but no ECG changes, as noted by Hirata et al. and Hirst et al. [4,5]. Eather et al. [6] published a systematic review of 18 cases of AAD being the cause of pericarditis and concluded that it more commonly occurs in young males and is associated with heterogeneous clinical findings, delaying diagnosis. This variability can result in missed diagnoses of AAD or lead to complications like pericardial effusion when pericarditis is not correctly identified. Saner et al. [2] provide further examples of this phenomenon in which early indications of pericarditis were present in five patients who experienced aortic dissection. Four patients had clinically apparent acute pericarditis, whereas the fifth patient was detected during autopsy. Additional case reports, as documented by Shariff et al. and Soyer [7,8] provide further examples of this diagnostic mimicry.

Our patient's persistent and worsening chest pain, later described as pruritic, prompted repeated assessments via ECG and cardiac enzyme levels. Initially, abnormal markers like elevated BNP levels were attributed to kidney dysfunction and elevated D-dimers to the AAD. However, subsequent ECGs showed widespread ST-segment elevation in leads V3-V6 and PR-segment depression in leads I, II, V4-V6 typical of acute pericarditis. The elevated CRP and ESR levels were likely associated with the inflammation in the pericardium, though these markers are non-specific. The elevated troponin levels suggested the possibility of coronary occlusion and myocardial ischemia. However, due to the patient's advanced age, the decision-maker declined coronary angiography. Despite TTE and CT chest not revealing pericardial changes, these diagnostic imaging methods can often be normal when pericardial thickness is <5 mm, and therefore, do not exclude the diagnosis of pericarditis [9]. The patient was empirically treated to prevent the development of pericardial effusion, alongside blood pressure management in the context of AAD. During the hospitalization, the patient's clinical improvement and the normalization of ECG findings and cardiac markers supported the diagnosis. His kidney function remained stable, indicating a likely underlying chronic condition.

This case raises the question of whether AAD was linked to the development of acute pericarditis or if it occurred coincidentally. In reports by Hirata et al., Hirst et al. and Eather et al. [4-6], pericarditis has been associated with ascending AAD. However, our patient had descending AAD, suggesting that these conditions were more likely coincidental and idiopathic in origin.

Acute pericarditis demonstrates infectious or non-infectious etiologies. Infectious etiologies range from viral to bacterial infections and can be associated with immunosuppression when pathogens are fungal or parasitic (Echinococcus, Toxoplasma). In non-infectious etiologies, the options are broad and include malignancy, auto-immune diseases (systemic lupus erythematosus, rheumatoid arthritis, Behcet disease), idiopathic causes (more common) and metabolic diseases (uremia and myxedema). Despite uremic pericarditis being a differential in our patient, this etiology was unlikely given that it more often manifests in end-stage kidney disease with BUN >60 mg/dL [10-12].

## Conclusions

This case highlights the rare and challenging presentation of AAD coexisting with acute pericarditis, emphasizing the critical need for clinicians to recognize and address these overlapping conditions promptly. Early identification and accurate diagnosis are essential to initiating appropriate treatment, as delays can lead to life-threatening complications. Furthermore, this case underscores the significance of adopting a multidisciplinary management approach, involving cardiology, cardiothoracic surgery, radiology, and emergency medicine teams, to ensure comprehensive care. It also serves as a reminder for healthcare providers to maintain a high index of suspicion, even in the absence of classic symptoms, as timely intervention can significantly improve patient outcomes and survival rates.
